# Xanthogranulomatous Cholecystitis: A Diagnostic Challenge for Radiologists, Surgeons, and Pathologists

**DOI:** 10.7759/cureus.10007

**Published:** 2020-08-25

**Authors:** Jawaria Rahman, Muhammad Tahir, Snehal Sonawane

**Affiliations:** 1 Pathology, Case Western Reserve University School of Medicine, Cleveland, USA; 2 Pathology, South Bend Medical Foundation, South Bend, USA

**Keywords:** gallstone cholecystitis, xanthogranulomatous cholecystitis, gall bladder carcinoma, acute cholecystitis, gall bladder histopathology

## Abstract

Xanthogranulomatous cholecystitis (XGC) is a rare inflammatory disease of the gallbladder characterized by severe proliferative fibrosis and the accumulation of lipid-laden macrophages in areas of destructive inflammation. Misdiagnosis is highly usual, and its macroscopic appearance may often be confused with gallbladder carcinoma. Here we discuss the case of a 56-year-old male who presented in the emergency room with fever, chills, and nausea. The routine laboratory investigations were normal except for elevated white blood cell counts. Abdominal ultrasound showed borderline gallbladder wall thickening. However, after CT scan findings, the suspect diagnosis of acute cholecystitis with possible perforation was made and the cholecystectomy was performed. The definitive diagnosis was delayed until the final pathology result came as a surprise, and later confirmed the histologic diagnosis of XGC. We consider this an important case because of the histopathologic finding of fibrotic thickened gallbladder wall with abundant histiocytes and pericholecystic fat stranding along with perforation and extensive inflammatory changes in the right upper quadrant of the abdomen which is highly suggestive and indicative of XGC in comparison to gallbladder carcinoma (GC). All things considered, clinically and grossly XGC presents in a similar fashion as GC; histopathology confirmed the diagnosis of XGC.

## Introduction

Xanthogranulomatous cholecystitis (XGC) is a remarkable inflammatory disease of the gallbladder that might be hard to distinguish from gall bladder malignancy [1]. In general, the overall occurrence of XGC is 1.3%-1.9%, except for India, where it is 8.8%. The frequency of gallbladder carcinoma (GC) related to XGC is less apparent in European studies (3.3%), differing from 5.1%-5.9% in the rest of the regions. Ambiguity with or undiscovered gall bladder carcinoma prompted 10.2% of patients getting over or under treatment [2]. Pathologically, XGC is described as inflammation with multiple intramural nodules [1]. The acute and chronic inflammatory cells, along with the intramural aggregation of lipid-laden macrophages, are the primary attribute of the disease [3,4]. Patients ordinarily present with manifestations and signs of acute cholecystitis. XGC is customarily a histopathological analysis of central or diffuse chronic and acute cholecystitis. Microscopically, lipid-containing histiocytes invading into the external layer of the muscle covering the gall bladder wall might be believed to form xanthogranulomatous foci and fibrosis inferable from extravasation of bile into the gallbladder wall [3,5]. Some of the cases of XGC are associated with gall bladder stones. Furthermore, XGC related fistulae to nearby organs and chronic inflammation add to the high pace replacement of laparoscopic into open procedures [6]. In this report, we have investigated the histopathological findings and imaging highlights of XGC to more readily comprehend the entity as well as to expand the diagnostic yield of the disease.

## Case presentation

A 56-year-old male presented to the emergency department with fever, chills, nausea, vomiting, and sharp, cramping pain in the right upper abdomen. He explained the pain as mild to moderate, continuous, and radiating to the back without association to food intake and alleviating or exacerbating factors. He denied any concern with bowel movements and urination. The signs for jaundice were negative. He admitted to a 15 lb weight loss over the past three months. The patient had a medical history of hypercholesterolemia and diabetes, for which he was taking oral medications; he had a history of smoking one pack of cigarettes per day per year for 30 years and drinking on social occasions. Past surgical history and family history were unremarkable. At the initial evaluation, his vital signs were within normal limits except the temperature of 37.8^o^C. Physical examination was significant for a firm, non-tender, grossly distended abdomen. A review of other systems was normal except for shortness of breath.

**Figure 1:**
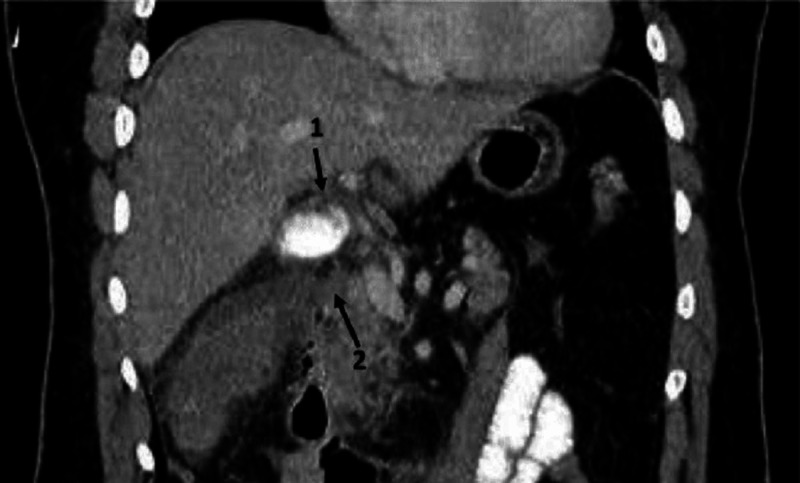
Coronal CT plane, showing extensive inflammatory changes and the thickening of gall bladder wall (1) with pericholecystic fat stranding (2)

The laboratory evaluation revealed normal levels of lactate, amylase, and lipase with an increase in total bilirubin and leukocytosis. Tumor markers, cancer antigen 19-9 (CA 19-9), and carcinoembryonic antigen (CAE) were in the normal range. The patient underwent abdominal ultrasonography (US), which showed borderline gallbladder wall thickening with multiple echogenic intramural foci suggestive of gallbladder adenomyomatosis. CT scan findings were suggestive of acute cholecystitis with possible perforation and showed extensive inflammatory changes in the right upper quadrant with gallbladder thickening and pericholecystic fat stranding (Figure 1). Endoscopic retrograde cholangiopancreatography did not show dilatation and any filling defects of the common bile ducts. After stabilization, the patient underwent cholecystectomy. Part of the gallbladder was submitted for frozen section.

**Figure 2:**
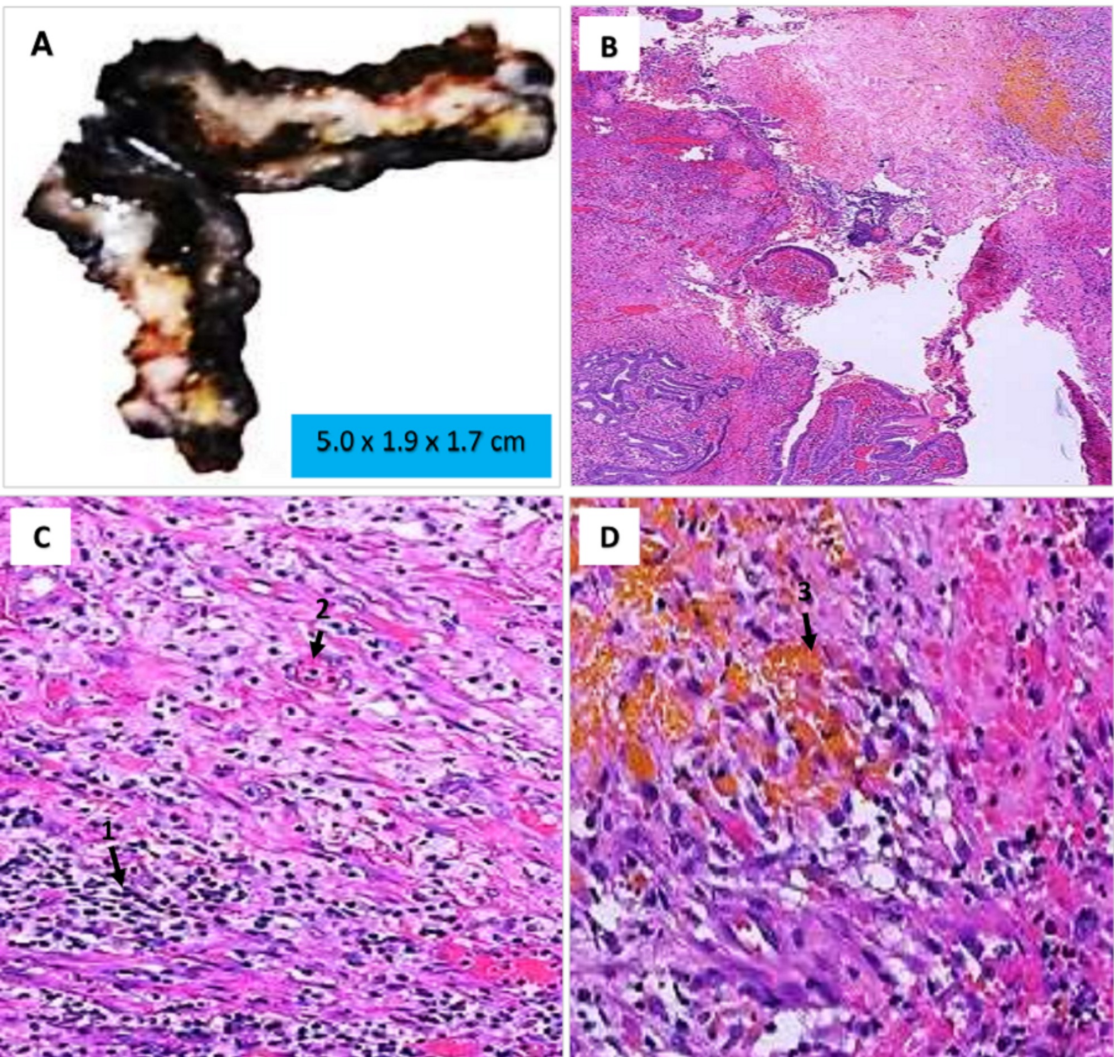
A: Gross image of the resected gallbladder with a thickened tan-white fibrotic wall concerning for adenomyomatosis or invasive process. B: Low power histologic appearance of gallbladder wall showing an ulcerated, and fibrotic wall. C: Medium power histologic appearance of gallbladder wall showing dense acute and chronic inflammation with abundant histiocytes (1) and giant cells (2). D: High power histologic appearance of gallbladder wall showing a chronic inflammation with histiocytes, macrophages and bile pigment dissipation (3).

On macroscopic examination, the resected gallbladder measured 5.0 x 1.9 x 1.7 cm, had serosal adhesions and a thickened wall with a tan-white fibrotic appearance concerning for adenomyomatosis, GC, or invasive process (Figure 2A). Choleliths were absent. Histologic examination showed an ulcerated, fibrotic gallbladder wall mucosa (Figure 2B). The abundant histiocytes and giant cell reaction were seen on high yield, as well as dense acute and chronic inflammation with bile dissipation (Figures 2C-2D). Evidence of dysplasia or malignancy were absent. A diagnosis of XGC was rendered.

## Discussion

XGC is an uncommon variation of cholecystitis depicted as “fibroxanthogranulomatous irritation” by Christensen and Ishak in 1977, and the Armed Forces Institute of Pathology later authored the term xanthogranulomatous cholecystitis [7]. XGC can be found in more than 9% of patients with cholecystectomy in India, 1.9% in Japan and Korea, 1.5% in America, and 1.3% in Europe [2]. Although we can see the clear geographic variation, we still could not find any contributing factor related to a specific region. The pathogenesis of XGC is not surely known, and it is speculated that inflammation of the gall bladder wall and surrounding area related to infection and impediment of biliary outflow bring about a burst of Rokitansky-Aschoff sinuses, which prompt mucosal ulceration and spilling of bile into the gallbladder wall. Histiocytic and multinucleated giant cells collect to immerse the bile, which later get adapted and provoke the fibrotic process [3]. Furthermore, the inflammation related to the xanthogranulomatous gallbladder can be extreme. It can extend to neighboring structures such as liver, bowel, and stomach, bringing about perforation, thick adhesions, abscess development, and a fistulous connection with an adjoining gut. Remarkable gallbladder wall thickening and dense nearby adhesions can be handily confused with carcinoma of the gallbladder [4]. Christensen and Ishak and Amazon and Rywlin noticed a pseudotumoral type of chronic cholecystitis that was described by the presence of xanthoma-like frothy cells and scarring and that comprised ceroid (wax-like) nodules in the wall of an inflamed gallbladder. The manifestations of XGC are typical of acute cholecystitis, including right upper quadrant pain, vomiting, obstructive jaundice, cholangitis, and a palpable mass [7,8]. Nevertheless, obstructive jaundice and cholangitis were not seen in our case. Inflammatory changes reaching the cystic duct and resulting in obstacles can clarify the development of increased bilirubin. Although, ongoing literature has indicated MRI is more precise than CT and high-resolution ultrasound in the determination of XGC but the presence of gallstones, diffuse thickening, and collapsed lumen are autonomously connected with XGC on high-resolution ultrasound. However, these discoveries can also be found in adenomyosis and carcinoma of the gallbladder [8]. Choleliths are generally present in XGC but were missing in our case. The characteristics of XGC like, adhesions, thickening of the gallbladder wall, and infiltration of pericholecystic fat can grossly imitate GC. Microscopic examination is the foundation for the diagnosis of XGC and shows a varying degree of inflammation-forming foamy histocytes, giant cells, and fibrosis with lacking dysplastic or malignant change [9].

Determining the exact finding in such confounding cases could be an overwhelming assignment, and it is typically deferred until the last pathology results. The trouble in characterizing the correct diagnosis and the methodology originates from the way that both XGC and GC share comparative clinical, imaging, and intraoperative characteristics. Despite the fact that some imaging discoveries on CT and US (for example, diffuse wall thickening, hypodense bands or intramural hypoattenuating nodules, gallstones, and ceaseless mucosal line) have been related more regularly with XGC in some studies [10,11].

Intraoperative frozen section biopsies or preoperative fine-needle aspiration plays a significant role and aid in differentiating the diagnosis and to the specialist in the decision-making process [11,12]. All these elements add up to extensive management along with diagnostic indeterminacy and can produce critical trouble in patients and patients’ families which may possibly result in imbalanced treatment [2]. However, the most common treatment of choice is simple cholecystectomy for a benign gallbladder such as XGC.

## Conclusions

XGC is difficult to diagnose either pre- or intraoperatively and remains challenging in clinical practice. Definitive diagnosis relies on the histopathologic assessment. Fine-needle aspiration cytology might be useful in impracticable cases. However, diagnosing atypical cases could be laborious, but recognizing the pathological changes found in the disease together with the spectrum of imaging discoveries can be valuable knowledge providing to the pathologists and radiologists.
